# Effect of Diatomite on the Thermal Degradation Behavior of Polypropylene and Formation of Graphene Products

**DOI:** 10.3390/polym14183764

**Published:** 2022-09-08

**Authors:** Yankun Chen, Biao Wang

**Affiliations:** State Key Laboratory for Modification of Chemical Fibers and Polymer Materials, College of Materials Science and Engineering, Donghua University, Shanghai 201620, China

**Keywords:** thermal degradation, polypropylene, diatomite, graphene, TG–FTIR, GC–MS, mechanism

## Abstract

In this work, the thermogravimetry–Fourier transform infrared spectroscopy (TG–FTIR) and gas chromatography–mass spectrometry (GC–MS) techniques are used to investigate the thermal degradation behavior of polypropylene (PP) with 20 wt.% diatomite (DM). The initial decomposition temperature of these blends was 17 °C lower than that of pristine PP, and more olefin degradation products were formed during the pyrolysis process under Ar atmosphere. These results could be attributed to the catalytic effects of DM on the degradation of PP and the changes of PP chain scission pathways around the particles (more β scission happened via the secondary radical transfer). These olefins could be caught by DM through the Si–O–C bond formed during the heat–treatment around 400~500 °C. The formation of the cross–linked structure could facilitate the growth of graphene during a high–temperature graphitization process.

## 1. Introduction

Graphene, an atomically thin two–dimensional material consisting of sp^2^–hybridized carbons, has attracted enormous attention in the scientific community, owing to its excellent physical, mechanical, optical, electrical, and thermal properties [1,2,3,4]. Many gas carbon sources such as methane (CH_4_) [5,6], ethylene (C_2_H_4_) [7,8], and acetylene (C_2_H_2_) [9,10] have been used to prepare graphene (flakes or sheets) by the chemical vapor deposition (CVD) method. The carbon atoms from the decomposition of these gas carbon sources dissolve in the metal substrates under argon or reductive atmosphere at high temperature, and then segregate and precipitate (nucleate and grow) into graphene during the cooling process [11,12,13,14]. When low–carbon–soluble metal or non–metal substrates were used, graphene with few layers was obtained through a self–limiting surface growth mechanism or a surface reaction mechanism [15,16,17,18,19,20,21]. In order to improve productivity and cut the cost, some solid carbon sources such as polypropylene (PP) [22,23], polyethylene (PE) [24], and polystyrene (PS) [25] have been applied to prepare graphene on the surface of metal or non–metal substrates. In these cases, the dissolution–segregation–precipitation mechanism and/or surface growth mechanism have also been used to elucidate the possible graphene growth mechanism during the solid–state CVD process.

However, solid carbon sources such as PP always have higher molecular weight, and the mechanism of degradation is much more complicated [26,27]. For example, CH_4_ mainly decomposed into carbon and hydrogen during heat treatment [28], while PP was more likely to be initially degraded into various hydrocarbons such as olefins [29]. Therefore, we think the mechanism of graphene growth must be different when the solid carbon sources are used, and the degradation products may affect the graphene formation during the pyrolysis process. Recently, Tang et al. found that the organically modified montmorillonite (non–metal substrate) could catalyze PP decomposition and thus affect the degradation products, which led to a high yield of graphene [22]. However, to our knowledge, the in–depth understanding of the formation mechanism of graphene during the pyrolysis of solid carbon sources (such as PP) is very limited and has not yet been systematically studied.

Polypropylene (PP) has been explored as an outstanding feedstock for pyrolysis production of high–valuable carbon materials by virtue of its high carbon content (85.7%), easy processability, and low cost [30,31,32,33,34,35]. The thermal degradation of PP is very complicated and occurs by random scission followed by the radical transfer process [36,37]. According to the pathways of PP chain cleavage to form radicals (primary and secondary radicals), α and β scission were put forward [38,39]. The type of the hydrocarbon product of α scission pathway is 3n (n = monomer unit), and that of a β scission pathway could be classified into three types: 3n, 3n + 1, and 3n + 2 [39]. These pathways often occur simultaneously, and the degradation products are greatly affected by the reaction temperature, residence time, and catalysts [40,41,42,43,44].

Recently, we successfully synthesized high–quality multilayer graphene using the diatomite/polypropylene (DM/PP) blends as the carbon feedstock via a one–pot pyrolysis method [45]. In this study, we focused on the formation of graphene during the pyrolysis process and explored the effect of DM on thermal degradation behaviour and degradation products of PP using the gas chromatography–mass spectrometry (GC–MS) and thermogravimetry–Fourier transform infrared spectroscopy (TG–FTIR) techniques. A novel possible mechanism of the formation of graphene during the thermal degradation of PP was proposed.

## 2. Materials and Methods

### 2.1. Materials

Pure PP pellets with a melt flow index (MFI) of 12 g/10 min were purchased from Jinshan Petroleum & Chemical Corporation (Shanghai, China). Raw diatomite powder with a median particle diameter of 22 μm (contained more than 85 wt.% SiO_2_), sulfuric acid (H_2_SO_4_, 98%), hydrofluoric acid (HF, 40%), nitric acid (HNO_3_, 65%), ethanol (95%), and acetone (99.5%) were provided by Sinopharm Chemical Reagent (Shanghai, China). Raw diatomite powder was purified with a mixture of nitric acid and sulfuric acid in a molar ratio of 2:1 to remove impurities [17]. The purification process of raw diatomite did not change the morphology and size of the diatomite skeleton, which was confirmed using scanning electron microscopy (SEM).

### 2.2. Methods

#### 2.2.1. Preparation of Diatomite/Polypropylene Blends

The DM/PP blends were prepared using the melt–mixing method in the micromixer (HLY–6/18–C5, Donghua University, Shanghai, China). The melt mixing speed, mixing temperature, and mixing time were 90 rpm, 230 °C, and 10 min, respectively. The DM/PP sample with DM content of 20 wt.% was prepared and designated as DM–20/PP.

#### 2.2.2. Graphene Synthesis by One–Pot Pyrolysis

The DM–20/PP pyrolysis experiment was carried out under argon with a flow rate of 200 sccm in a quartz tube placed in a tube furnace (50 × 700/10 K − 26 C, Shanghai Yifeng Electrical Furnace Co., Ltd., Shanghai, China). DM–20/PP was heated from room temperature to 1000 °C (pyrolysis temperature) with 100 min (holding time) at 20 °C min^−1^, and then the furnace was cooled to room temperature in argon. The cooled residue was collected as the initial pyrolysis products of DM–20/PP, and this residue was then immersed in HF solution with a molar ratio of HF: H_2_O: ethanol = 7:30:5 for 12h at room temperature to eliminate DM, amorphous carbon, and additives. After centrifugation with ethanol and water, and then drying, the purified pyrolysis products (graphene) were obtained. Further information on the one-pot pyrolysis method is detailed in our previous study [46].

#### 2.2.3. Characterization

##### Morphology and Microstructure of Graphene

The morphology and microstructure of the obtained graphene were observed by transmission electron microscopy (JEOL, JEM–2100–TEM, Tokyo, Japan). For the TEM measurements, a small amount of graphene powder sample was evenly dispersed in ethanol, and then the dispersion was drop cast onto a holey carbon film supported on a copper grid. Raman spectroscopy was performed by the laser Raman spectrometer (inVia Reflex, Renishaw, Gloucestershire, UK) using an excitation beam wavelength of 532 nm. Powder X-ray diffraction (XRD) was carried out by a DMSX–2500 PC X-ray spectrometer with Cu Kα radiation (λ = 0.154 nm) operating at 40 kV and 35 mA.

##### TG–FTIR Analysis

Thermogravimetry–Fourier transform infrared spectroscopy (TG–FTIR) analysis was performed using a thermogravimetric analyzer (TGA 209F1, Netzsch, Selb, Germany) coupled with an FTIR spectrophotometer (Nicolet Nexus 6700, Bruker, Bremen, Germany) by a Thermo–Nicolet TGA special connector. The stainless–steel transfer pipe and gas cell were heated at 200 °C. The real reaction temperature was precisely controlled by the programmed temperature controller. The samples were heated from 40 °C to 1000 °C with a heating rate of 20 °C min^−1^ under N_2_ atmosphere with a constant flow rate of 20 mL/min at atmospheric pressure. Resolution in FTIR spectrum was set at 4 cm^−1^, with a scan frequency at 20 times per minute, and the spectral region at 4000–400 cm^−1^.

##### GC–MS Analysis

Gas chromatography–mass spectrometry (GC–MS) analysis was carried out on a GC–MS–QP2010 spectrometer (Shimadzu, Kyoto, Japan) equipped with a fused silica capillary column for the analysis of the degradation products. The temperature program was set to an initial oven temperature of 50 °C, and was increased at a rate of 20 °C min^−1^ to 700 °C using helium as a carrier gas. The profiles of products with different retention times were gained and the background noise was subtracted. The mass spectrometry of degradation products in samples were obtained by electron ionization at 70 eV, and the data was evaluated by employing total ion count for product identification and quantification. Quantitative analysis of pyrolysis products was performed by the area normalization method [46].

## 3. Results and Discussion

### 3.1. Characterizations of Morphology and Structure of Graphene

**Figure 1:**
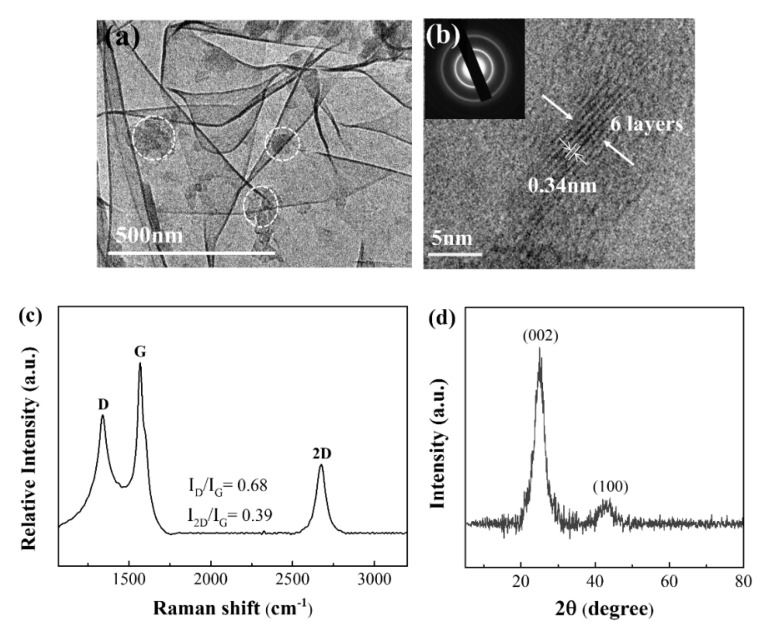
(**a**) Transmission electron microscopy (TEM ) image and (**b**) High resolution transmission electron microscope (HRTEM) image of as–prepared graphene by pyrolysis of Polypropylene (PP) with 20 wt.% Diatomite (DM–20/PP). The inset in (**b**) shows the Selected area electron diffraction (SAED) pattern of graphene layers. (**c**) Raman spectra and (**d**) X-ray diffraction (XRD) pattern of the as-prepared graphene by pyrolysis of DM–20/PP.

According to our previous study [45], high–quality graphene with 4–6 layers was obtained using DM/PP as the carbon feedstock via the one–pot method under the pyrolysis time of 100 min and pyrolysis temperature of 1000 °C. Figure 1a shows the TEM image of graphene prepared from pyrolysis of PP with 20 wt.% DM. The transparent wrinkled graphene platelets with curved edges and some non–etched DM (circled in Figure 1a) were observed. The high–resolution TEM (HRTEM) image of the obtained graphene clearly shows the presence of graphene layers, and the distance between these graphene layers was about 0.34 nm (arrows in Figure 1b). The selected area electron diffraction (SAED) pattern of these layers shows a typical ring–like pattern indicating the polycrystalline nature of the as–prepared graphene (upper inset in Figure 1b) [47]. The Raman spectra of graphene (Figure 1c) revealed three obvious sharp peaks located at 1340, 1568, and 2674 cm^−1^, which corresponded to the D band, G band, and 2D band, respectively. The I_D_/I_G_ intensity ratio of 0.68 and I_2D_/I_G_ intensity ratio of 0.39 implied the presence of few–layer graphene with a high graphitization degree and low contents of structural defects [48]. The XRD analysis was also performed on the as-prepared graphene, as shown in Figure 1d. The XRD pattern displays diffraction peaks at 25.8° and 42.3°, which are assigned to the (002) plane and (100) plane, respectively. These peaks corresponded to the hexagonal graphite structure and the combination of turbostratic graphite and crystalline graphite, respectively [49]. The d-spacing value of the (002) peak corresponds to an interlayer spacing of 0.344 nm, which is in good accordance with the HRTEM result. The average number of graphene layers per stack (n) could be calculated by the Bragg equation and Scherrer formula [50,51]. The value n of the synthesized graphene was about 5.9, indicating the as–prepared graphene with less than 10 layers formed during the pyrolysis process [51]. This result is in good agreement with the HRTEM and Raman results (Figure 1b,c).

### 3.2. Effect of DM on the Thermal Degradation Behavior of PP

**Figure 2:**
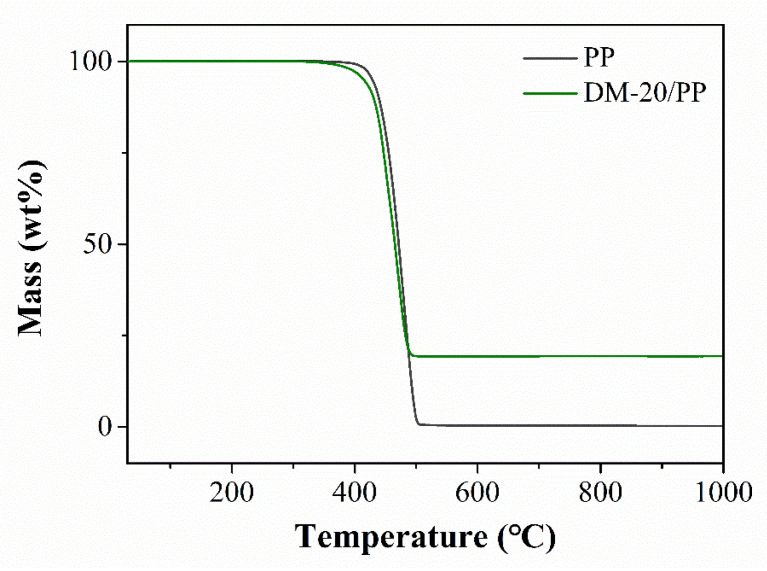
Thermogravimetric analyzer (TG) curves of pure PP and DM–20/PP.

The decomposition temperature of thermoplastic polymers is always increased when inorganic particles are filled [52,53,54]. This phenomenon is mainly attributed to the limited behavior of molecules around the particles (especially the nanoparticle fillers with the higher specific surface area) [55,56,57]. Interestingly, we found that the initial decomposition temperature (obtained by the temperature corresponding to the 5% mass loss of the sample) of PP with 20 wt.% DM was 412 °C, 17 °C lower than that of pristine PP (Figure 2), indicating that the DM could catalyze the thermal degradation of PP.

**Figure 3:**
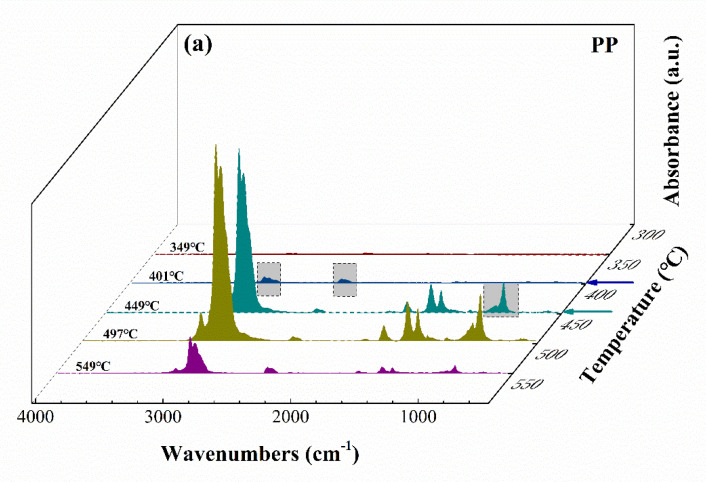

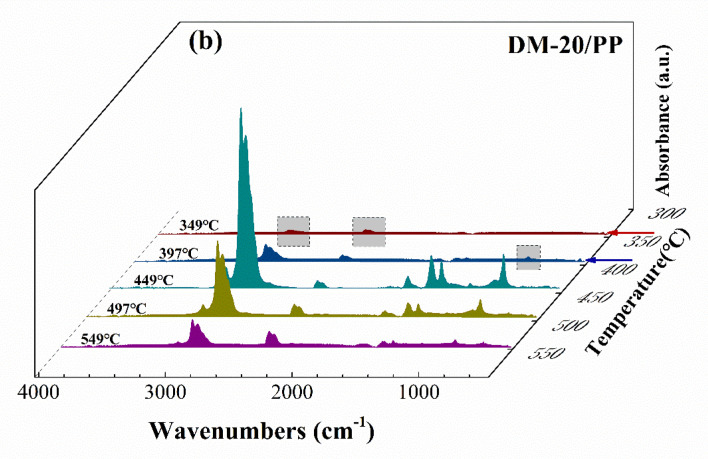
Three-dimensional TG–FTIR diagrams of pyrolysis products of (**a**) pure PP and (**b**) DM–20/PP.

Thermogravimetry–Fourier transform infrared spectroscopy (TG–FTIR) diagrams of PP and DM–20/PP are shown in Figure 3. From Figure 3a, the main infrared absorption peaks of the pure PP sample at 880~990 cm^−1^, 1150~1470 cm^−1^, 1640~1660 cm^−1^, 2310~2360 cm^−1^, 2880~2930 cm^−1^, and 2950~2970 cm^−1^ were observed, which appeared at 449 °C, 449 °C, 449 °C, 401 °C, 401 °C, and 401 °C, respectively. These main IR peaks corresponded to the =C−H, −C−H, and C=C bond, and CO_2_, −CH_2_, and −CH_3_ groups of various pyrolysis products of PP, respectively. When PP was incorporated with DM, the positions of these absorption peaks did not change, while the temperature of the appearance of some peaks moved ahead (Figure 3b). For example, for DM–20/PP, the peaks at 880~990 cm^−1^, 2310~2360 cm^−1^, and 2950~2970 cm^−1^ appeared at the temperature of 397 °C, 349 °C, and 349 °C, respectively. The appearance temperatures of these peaks were all 52 °C lower than those for the PP sample (arrows in Figure 3), indicating the degradation products of DM–20/PP formed ahead. This result was in good agreement with the TG results, and further confirmed that the catalytic effect of DM on the thermal degradation of PP due to DM could reduce the degradation activation energy of PP [26,58].

**Figure 4:**
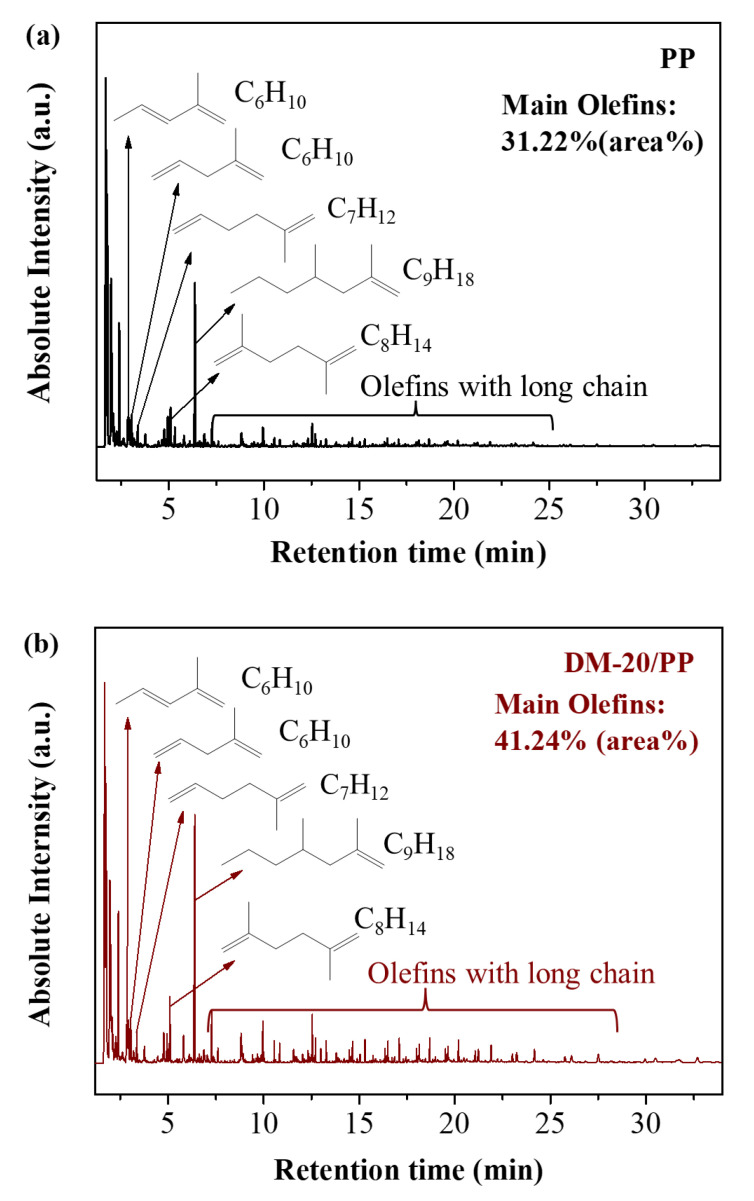
GC–MS chromatograms for the main olefins evolved from the thermal degradation of (**a**) PP and (**b**) DM–20/PP.

The GC–MS measurement was performed to further investigate the influence of DM on the categories and quantities of pyrolysis products of PP. Table 1 displays the content of main degradation products of pure PP and DM–20/PP, and the detailed results of the total components of products are listed in Table S1. Although the main degradation products of DM–20/PP were the same as those of PP, the quantity of these products was very different. Among these products, the quantities of the main olefins (>C_5_) increased. Figure 4 shows that the GC–MS chromatograms of the main olefins evolved from PP and DM–20/PP during the thermal degradation. The total yield of these main olefins’ products from DM–20/PP, including 1–pentene, 2,4–dimethyl–1–heptene, 2,5–dimethyl–1,5–hexadiene, 2–methyl–1,4–pentadiene, trans–2–methyl–1,3–pentadiene, 2–methyl–1,5–hexadiene and olefins with the long chains, was about 41.24% (area%), 32% higher than that from PP (31.22%, area%). This result indicated that DM not only promoted the thermal degradation of PP, but also increased the yield of olefins products during the pyrolysis process. The product type of these main olefins could be classified into three types: 3n, 3n + 1, and 3n + 2 (n = monomer unit). These three types of pyrolysis products could be ascribed to the β scission pathway of PP [29,39]. Therefore, we think that the DM could promote chain cleavage of PP via more β scission around DM particles, leading to the generation of more olefin products.

**Figure 5:**
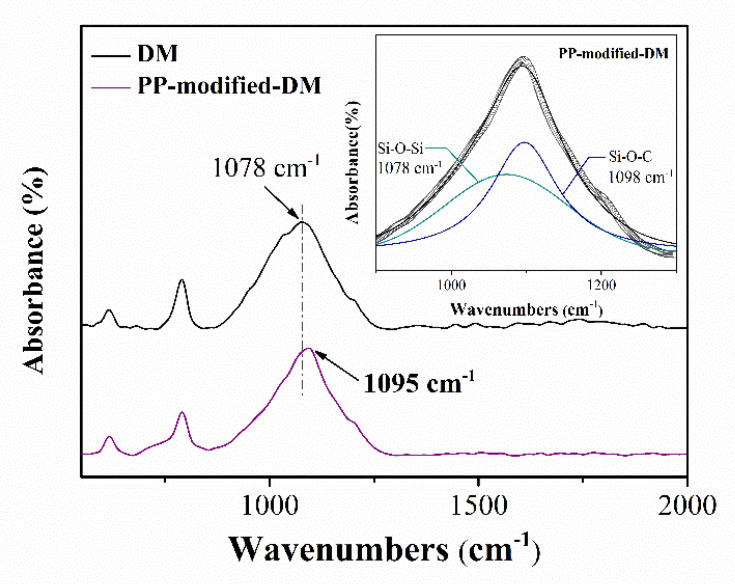
FTIR spectra of PP–modified–DM and DM samples after heat treatment.

In order to explore the interaction between DM and pyrolysis products during the heat treatment, a heat–treated sample was prepared by pyrolysis of PP–modified–DM at 450 °C for 5 min under Ar, and then FTIR measurement was carried out. The PP–modified−DM was prepared by impregnating DM into 2.2 wt.% PP methylbenzene solution, followed by filtration and drying. The distinct absorption peak at 1078 cm^−1^ of DM originates from the asymmetric stretching vibration of Si−O−Si, as shown in Figure 5. However, for PP–modified–DM, the absorption peak shifts to 1095 cm^−1^. This peak was deconvoluted to two different peaks, which corresponded to the vibration of the Si−O−C bond at 1098 cm^−1^ and the Si−O−Si bond at 1078 cm^−1^. This result confirmed that DM was involved in the reaction with the pyrolysis products through the formation of the Si−O−C bond, and DM catches the pyrolysis products to prevent them being carried away by the carrier gas. The thermal degradation of PP could generate radicals of olefins via cleavage of the PP chain and radical transfer. Therefore, we think these radicals in olefins are most likely to react with DM particles during the heat treatment.

### 3.3. Discussion about the Possible Mechanism of Graphene Formation

**Figure 6:**
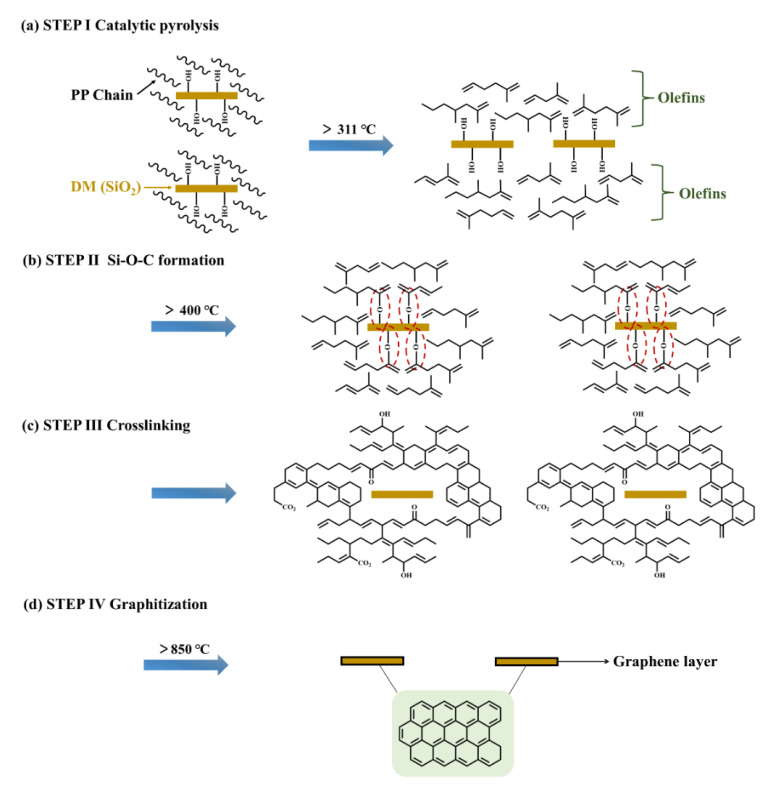
Schematic of the possible mechanism of graphene growth during the pyrolysis process.

On the basis of the above results, the growth mechanism of graphene by pyrolysis of DM–20/PP is different from graphene prepared by the CVD method using gas carbon sources. We propose a novel possible mechanism to explain the formation of graphene using PP as the solid carbon source, which consists of the following steps (Figure 6): (i) DM catalyzed the thermal degradation of PP and more β scission occurred via the secondary radical transfer around the DM particles, leading to the higher yield of main olefins’ products (Figure 6a). (ii) These olefins could react with DM through the Si–O–C bond formed during the heat treatment around 400~500 °C (Figure 6b). (iii) A cross–linked infusible structure could form under higher temperature (Figure 6c). (iv) This cross–linked infusible structure could facilitate the growth of graphene during a high–temperature graphitization process (Figure 6d) [59,60].

## 4. Conclusions

High-quality graphene with few layers was synthesized using the diatomite/polypropylene (DM/PP) blends as the carbon feedstock via a one-pot pyrolysis method. DM plays a catalytic role in the thermal degradation of PP, and thus enables the initial decomposition temperature to decrease and the olefin products to increase. Meanwhile, DM is involved in the reaction with the pyrolysis products through the formation of the Si−O−C bond during the heat treatment at around 400~500 °C. The cross-linked infusible structure could form around the DM surface, and then promote the growth of graphene during a high-temperature graphitization process.

## Figures and Tables

**Table 1:** The content of main degradation products of pure PP and DM–20/PP.

Main Degradation Products	PP Sample (Area%) ^1^	DM–20/PP Sample (Area%) ^1^
Propylene	28.72	24.56
2–Methyl–propene	14.86	13.19
1–Pentene	9.31	11.35
2–Methyl–1,4–pentadiene	2.00	2.06
trans–2–Methyl–1,3–pentadiene	1.40	1.59
2–Methyl–1,5–hexadiene	1.08	1.26
2,5–Dimethyl–1,5–hexadiene	2.16	2.58
2,4–Dimethyl–1–heptene	8.70	11.41

^1^ Calculated by Gas Chromatography–Mass Spectrometry (GC–MS) analysis.

## Data Availability

Data are contained within the article.

## Supplementary Materials

The following supporting information can be downloaded at: https://www.mdpi.com/article/10.3390/polym14183764/s1, Table S1: The normalized peak areas of the evolved products during thermal degradation of pure PP and DM–20/PP samples according to Figure 4.
