# Supplementary material for: Effect of Diatomite on the Thermal Degradation Behavior of Polypropylene and Formation of Graphene Products
Source: Polymers (Basel). 2022 Sep 8;14(18):3764. doi: 10.3390/polym14183764 (PMC9501155; doi:10.3390/polym14183764)
Supplement: Supplementary file 1 [file polymers-14-03764-s001.zip › polymers-1912941-supplementary.pdf]

Supporting Information

Effect of diatomite on the thermal degradation behavior of polypropylene and formation of graphene products

Yankun Chen [1] and Biao Wang [1,*]

[1] State Key Laboratory for Modification of Chemical Fibers and Polymer Materials, College of Materials Science and Engineering, Donghua University, Shanghai 201620, China.
*Corresponding author: Biao Wang, E-mail: wbiao2000@dhu.edu.cn

Results and discussions Section

Table S1. The normalized peak areas of the evolved products during thermal degradation of pure PP and DM-20/PP samples according to the Figure 4.

Pyrolysis products	Molecular Formula	PP sample (area%) [1]	DM-20/PP sample (area%) [1]
Propylene	C3H6	28.72	24.56
2-Methyl-Propene	C4H8	14.86	13.19
1,3-Pentadiene	C5H8	10.59	8.86
1-Pentene	C5H10	9.31	11.35
2-Methyl-1,4-pentadiene	C6H10	2.00	2.06
trans-2-Methyl-1,3-pentadiene	C6H10	1.40	1.59
1-Hexene	C6H12	10.05	8.60
1,3,5-Cycloheptatriene	C7H8	1.67	-
2-Methyl-1,5-hexadiene	C7H12	1.08	1.26
2,4-Dimethyl-1-pentene	C7H14	1.46	1.48
1,3-Dimethylbenzene	C8H10	0.89	-
2,5-Dimethyl-1,5-hexadiene	C8H14	2.16	2.58
2-Methyl-1-heptene	C8H16	0.87	-
1,7-Nonadiene	C9H16	-	0.48
2,4-Dimethyl-1-heptene	C9H18	8.70	11.41
1-Decanol	C10H22O	0.34	1.54
2-Decene	C10H20	0.31	1.44
4-Methyl-2-decene	C11H22	1.51	1.94
1-Dodecanol	C12H26O	0.46	1.53
7-Methyl-1-undecene	C12H24	1.07	1.64

4-Tetradecene	$C_{14}H_{28}$	0.30	1.42
4-Isopropyl-1,7-dimethylcyclodecane	$C_{15}H_{30}$	0.33	-
3-Eicosene	$C_{20}H_{40}$	1.92	3.07

[1] Calculated by GC-MS.
